# Fellow-Workers and the Roll of Honour

**Published:** 1915-12-04

**Authors:** 


### ROUND THE WORLD.

**[The Editor invites Early Information and Contributions Marked "Fellow-Workers."]**

### Fellow-Workers and the Roll of Honour.

Lieut.-Colonel Alexander Aitkin Ross, R.A.M.C., 3rd Midland Field Ambulance (T.F.), died of wounds on November 24 at an officers' hospital in London. His name has not up to the present appeared in the casualty lists, but his death was recorded in the *Times* on November 26. He was the son of the late Lieut.-Colonel A. A. Ross, of the Leicestershire Regiment, and obtained his M.B. degree at Edinburgh University in 1893. He had been in command of the 3rd Lowland Field Ambulance since August 1908.

Lieutenant J. C. Bell, R.A.M.C., attached 7th Royal Scots (T.F.), who was on service with the Mediterranean Expeditionary Force, died of dysentery in hospital at Alexandria on November 22. Lieutenant Bell obtained his M.R.C.S., L.R.C.P. in 1900, having studied at University College Hospital. For a long period he was in practice at West Coker, Somerset. He obtained his commission in December last.

Lieut. and Quartermaster H. Robinson, R.A.M.C., who has died on service, was born in 1876. After some eighteen years in the ranks, he was appointed sergeant-major in the R.A.M.C. in August 1914, and was given his commission in June this year.

Lieutenant J. R. Spensley, R.A.M.C., attached 8th Buffs (East Kent), who was previously reported killed, is now reported wounded and a prisoner of war. Lieut. Spensley became an M.R.C.S., L.R.C.P. in 1891; prior to the war his registered address was in Genoa, Italy, but he was formerly a house surgeon at the London Hospital. He became a temporary lieutenant in the R.A.M.C. in May last.

Lieutenant F. T. Sampson, R.A.M.C., attached 2nd Dorset Regiment, who has been serving in the Persian Gulf, is reported wounded. He obtained his M.B. degree at the University of Durham in 1906. At the outbreak of war he was in India, but prior to that he lived at Shipston-on-Stour, Worcestershire.

Captain R. W. S. Murray, M.B., R.A.M.C., who is reported wounded, obtained his degree at Aberdeen University in 1912. When the war broke out he was house surgeon at the General Hospital, Tunbridge Wells.

Captain Arthur Martin Leake, V.C., F.R.C.S., has been gazetted as a temporary major, R.A.M.C. It will be remembered that Major Leake, who gained the Victoria Cross in 1902, was awarded the clasp in February this year for conspicuous bravery and devotion to duty in the present campaign.

Lieut.-Colonel Richard Heard, who has been rewarded for his services in India by being promoted Brevet-Colonel, is Railway Medical Transport Officer at Bombay. He obtained his M.B. degree at Belfast in 1892, and the M.D. degree in 1912. At one time he held a resident appointment at the Adelaide Hospital, Dublin.

Dr. G. Landsborough Findlay, who has been rendering medical help to the Serbians, is reported to have arrived safely at Salonica with his wife, Lady Blanche Sybil Findlay, and a party of eight English doctors and sixteen English nurses. According to an account written for the United Press of America by Mr. William G. Shepheard, and published in some of the London newspapers, the party had trudged on foot for seven days through the Albanian mountains, along paths buried in snow and mud. They were short of supplies from the start, and fifteen of their twenty pack mules died of hunger on the way. Dr. Landsborough Findlay obtained his M.B. degree at Edinburgh University in 1897, and at one time held a resident appointment at the Royal Edinburgh Asylum. Lady Sybil Findlay, who shared her husband's privations, is the daughter of the Earl of Kingston.

Dr. Andrew M'Gregor Sinclair has been elected Mayor of Burnley. Dr. Sinclair went to Bolton in 1890 as the first resident medical officer of the Victoria Hospital, of which he is now honorary surgeon. He is also consulting surgeon to the Colne Cottage Hospital. Dr. Sinclair is well known throughout East Lancashire as a consultant and private practitioner. He obtained the M.B. degree at Aberdeen University in 1890.

The list of casualties reported as a result of the sinking of the hospital ship *Anglia* contains the names of one nurse—Miss M. Rodwell, Queen Alexandra's Imperial Military Nursing Service Reserve—and one corporal and eight privates of the R.A.M.C.

